# Catalytic abatement of CO species from incomplete combustion of solid fuels used in domestic cooking

**DOI:** 10.1016/j.heliyon.2018.e00748

**Published:** 2018-08-23

**Authors:** D.O. Obada, M. Peter, D.M. Kulla, N.O. Omisanya, A.Y. Atta, D. Dodoo-Arhin

**Affiliations:** a Department of Mechanical Engineering, Ahmadu Bello University, Zaria, Nigeria; b National Automotive Design and Development Council, Zaria, Nigeria; c Department of Chemical Engineering, Ahmadu Bello University, Zaria, Nigeria; d Department of Materials Science and Engineering, University of Ghana, Legon, Ghana; e Institute of Applied Science and Technology, University of Ghana, Legon, Ghana

**Keywords:** Energy, Materials science

## Abstract

This study reveals a first time approach to catalytic based interventions primarily on indoor air pollution emanating from commercial and household solid fuel burning in a region in Nigeria. An intensive survey of the temperatures at different locations in the common stoves used for cooking was conducted so as to ascertain temperatures suitable for catalyst efficiency and stability. Furthermore, cobalt and iron based catalysts were prepared using ultra stable Y type zeolite as supports. The synthesized catalysts were characterized for its physico-chemical properties. The catalytic efficiency of the supported catalysts was tested using simulated exhaust gases in a fix bed reactor. The study further explored real time testing of the catalyzed ceramic monolith using two different wood species. First, the best catalyst in terms of simulated exhaust testing was selected. Consequently, a small layer of zeolite Y was deposited at 3% of the monolith weight to enhance the subsequent adhesion of the best catalyst powder to the structured monolith. Then to catalyze the zeolite Y wash-coated monolith with the cobalt precursor, the dip coating technique was used. From the results, the average values of temperatures observed from the surveyed cook stoves using wood and plant residue as fuel were confirmed to be in the range of 203–425 °C which is considered suitable for catalysts activity. The Co/ZY catalyst showed approximately 100% CO conversion (T_100_) at 250 °C for initial CO concentration of 1000 ppm, making it the most effective, while T_100_ was increased to 275 °C and 325 °C for Fe/ZY and Co-Fe/ZY catalyst respectively at an exhaust residence time of 20000 h^−1^. The catalytic converter in real time testing for CO abatement performed well for both wood species. Only minor differences have been noticed.

## 1 Introduction

It has been identified that poor air quality was and is still a problem indoors, which has resulted in measures to reduce this problem through the provision of fresh air in the rooms, buildings etc (Matson and Sherman, 2004). In most of these scenarios, a complete combustion of fuels which contain only carbon, hydrogen, and oxygen should only produce CO_2_ and H_2_O. Nonetheless, in actual sense, this is almost impossible, as the stoves we use in our homes are not ideal combustion devices. In addition, the oxygen available for assisted combustion is deficient because of the solid nature of the fuels. This situation encourages the production of noxious pollutants such as carbon monoxide (CO), volatile organic compounds (VOCs), etc. Carbon monoxide has over the years been hugely released from incomplete combustion of these solid fuels (Gordon et al., 2004). It has been reported that an average of around 2.5 billion people constantly prepare their meals using solid fuels such as wood species, residues of plant, charcoal etc. It is pertinent to state that emissions from stoves using these solid fuels are disadvantageous to the climate, the quality of air inside our buildings, and related health effects (Alex et al., 2016).

Catalysts which are based on mixed oxides consisting prominently of transition metals are promising for many reactions which have huge environmental significance (Li et al., 2008; Morales et al., 2006, 2008; Du et al., 2008) and the control of noxious emissions in these stoves during operation is no exception. Usually, the catalysts itself are most often a mix of noble metals and are very expensive (Martin et al., 2002). The catalysts also have hydrothermal and chemical stability issues. From the economic and practical point of view, especially for developing countries like Nigeria, researchers are looking into a series of low-cost metals (non-noble metal based catalysts) which can be as catalytically active as their noble-metal counterparts without compromising catalytic properties.

Usually, catalytic converters used as retrofits (Fine et al., 2004; Ozil et al., 2009; Kaivosoja et al., 2012) are viable for mitigation of noxious emissions. This stems from the fact that as compared to other devices like filters, catalytic converters have this reductive influence on both gases and solid emissions (Bindig et al., 2012; Hukkanen et al., 2012; Bensaid et al., 2012).

Two very popular and very well distributed wood species in northern Nigeria known for domestic cooking are –*Delonix regia* and *Cassia siamea*. *Delonix regia* is a semi-deciduous tree referred to as flame of forest in Nigeria and can grow to heights of about 18 metres. *Cassia siamea* (Lam.) was introduced into Nigeria in 1889. It is cost effective to set-up if planted directly into the plantation site. As much as 136,100 kilograms of dry wood can be obtained in ten years (Chow and Lucas, 2007).

The emission of pollutants from these wood-fired stoves for domestic cooking is currently the object of an increasing concern for several researchers. Doggali et al. (2011) in their work designed Cu-Mn based mixed oxide type catalysts in supported form using mesoporous materials. The catalytic activity for CO was investigated from solid fuel burning using rural cook stoves. The trend observed for the catalytic activity of the synthesized catalysts for CO oxidation showed that supported ZrO_2_ delivered the best performance. Zhang et al. (2016) studied copper-ceria catalysts which were supported on a series of zeolites for CO oxidation. The findings revealed that the catalysts activity were influenced by the type of zeolite and the synthesis methods. Hukkanen et al. (2012) in their study investigated a wood stove incorporated with a catalytic converter as a secondary emission reduction plan and made comparisons with and without the catalysts. With the catalytic converter, CO reductions of up to 21% were achieved. Ozil et al. (2009) conducted characterization of pollutants from two catalytic converters which were placed in the exhaust of two domestic fireplaces (older and newer generation). Much more reduced concentration of CO in the exhaust was obtained with the new generation fireplace comparatively. Bensaid et al. (2012) fabricated a suitable after-treatment for the reduction of pollutant emissions from wood fired stoves. A series of perovskite catalysts was fabricated to conduct these experiments. The monolith coated with the selected catalysts was able to reduce all gaseous emissions throughout the wood burning cycle even at lowest temperature of around 200 °C. Doggali et al. (2015) investigated the catalytic properties of PrMnO_3_ and Ba/Ke/Ce substituted variants of the original catalyst for CO oxidation with a view to applying in rural based cook stoves. The findings suggested that the promotion effect of an alkali metal (K) on the “A” site of the catalyst reduced the activity of the catalyst towards CO oxidation. However, the substitution of Ba and Ce in the catalyst increased the CO oxidation process. Obada et al. (2016) in their study performed a structural and textural characterization to investigate the adherence of zeolite-based catalyst washcoated onto honey-comb type cordierite monoliths. It was concluded that the composite materials which were ZSM-5 and precursors of the transition salts of copper, zinc and ceria powders were successfully deposited on the catalyst supports. Reichert et al. (2018) in their work investigated the emissions from two types of noble-metal based honeycomb shaped catalyst monoliths incorporated into different stoves using solid fuel (firewood). They observed that the ceramic catalyst enhanced the reduction of CO emissions by 83%. Still et al. (2018), noted in their work that if cook-stoves are operated indoors and smoke is drawn through suction by a fan before been released to the outside, the overall pollution rates could be reduced. This method is practically possible for harmful emission reduction and could be enhanced with some catalytic technologies.

Despite increasing focus on the detrimental impacts of cooking with biomass, research efforts to find long lasting solutions are facing some challenges. These slight blips include matching stove technologies to the combustion characteristics of fuel used. In this way, to the best of our knowledge, the progression of the application of these efficient catalysts reported vis-à-vis the combustion characteristics of specific wood species have been rarely highlighted to ensure some compatible wood-catalyst synergies.

Hence, the aim of this study is to investigate the catalytic abatement of CO species from the non-negligible amount of incomplete combustion products from specific wood species commonly used for domestic cooking in northern Nigeria through the development of suitable catalytic composition and their application in whole-scale monoliths for implementation in real time testing.

## 2 Experimental

A survey was conducted in randomly selected 50 commercial and household locations for data collection on temperature of various parts of the stoves during operation with two variants of solid fuel. The target locations were points that use biomass fuels (wood and crop residue) as their main sources of cooking fuel. K-type thermocouple (Kane- May KM340, −50 °C ≈ 1500 °C) was used to collect data of the inner walls of the traditional cook stoves during these measurements. The thermocouples which were manually operated were logged at an interval of 30 secs at the various points of measurement.

Aqueous solutions of the calculated stoichiometric quantity of Co(NO_3_)_2_ and Fe(NO_3_)_3_ were used to synthesize metal solutions using deionized water. Catalysts were prepared by loading of 15 wt.% Co and 15 wt.% Fe on zeolite supports. For the mixed metal oxide loading, 7.5 wt.% Co and 7.5 wt.% Fe was targeted. Catalysts were prepared by the excess-solution impregnation method. Zeolites were pre-treated at 550 °C for 3 h in air to remove any adsorbed moisture and to transform the zeolite to its hydrogen form (H-ZY). For the synthesis procedure in terms of the weight % of metal oxide precursors, 22.0 g Co(NO_3_)_3_·6H_2_O and 7.0 g Fe(NO_3_)_3_·3H_2_O based on stoichiometric conditions were dissolved into 100.0 mL deionized water to prepare separate solutions for Co/ZY, Fe/ZY and Co-Fe/ZY catalysts. 5 g zeolites were added into each solution under stirring (10 min) and further impregnated with the metal oxide solution initially at room temperature before the temperature of solution was systematically increased as catalyst precursors were mixed to dryness. Finally, all as-prepared precursors were dried at 100 °C for 2 h and calcined at 550 °C in air for 3 h.

Chemical analysis of parent material (zeolites) and prepared catalysts (metal loaded zeolites) was performed using a Phillips PW2400 X-ray Fluorescence (XRF) machine. X-ray diffraction (XRD) data was recorded at room temperature on an empyrean diffractometer (Bruker AXS, D8 Advance) with theta/theta geometry, operating a Cu Kα radiation tube at 40 kV and 30 mA. The microstructure of the samples' crystal aggregates to reveal the size and morphology of the oxide crystals was determined using JEOL JSM-6380A scanning electron microscope equipped with an energy dispersive X-ray spectrometer (EDX).

Catalytic activity of all the synthesized catalysts was investigated for CO oxidation using a fixed bed reactor. The reactant gas feed contained 1000 ppm CO, 10% O_2_ and N_2_ balance and catalytic activity was subsequently measured at a simulated exhaust gas residence time of 20,000 h^−1^ using gas chromatograph. Based on investigations after successful synthesis of the catalysts, the best catalyst for CO oxidation was selected and anchored on ceramic supports. Cordierite honeycombs (diameter: 85 mm, length: 100 mm, cell density: 400 cpsi) was used as support materials. First, the best catalyst in terms of simulated exhaust testing was selected. Consequently, a small layer of zeolite Y was deposited at 3% of the monolith weight to enhance the subsequent adhesion of the best catalyst powder to the structured monolith. Then to catalyze the zeolite Y wash-coated monolith with the cobalt precursor, the dip coating technique was used. The specific amount of deposited catalyst precursor was 5% of monolith weight. Air was blown through the channels to avoid the formation of meniscus and dried in a hot air oven before calcination at 550 °C for 3 h. In a previous study (Kulla, 2011), a fabricated stove (Model 4 type) was observed to produce the best performance in terms of variables tested (percentage heat utilization, cooking efficiency etc), hence the need to investigate emission performance by incorporating the supported catalysts.

**Fig. 1:**
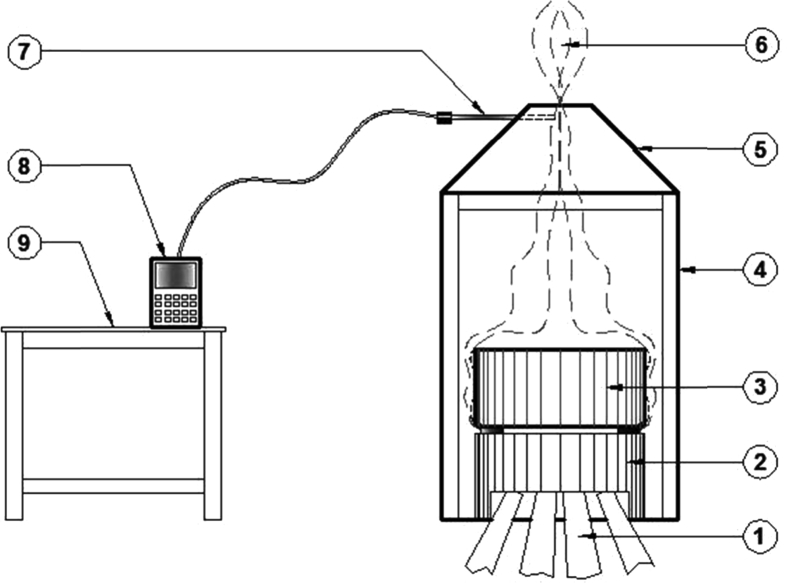
Schematic for emission testing using the extraction booth. (1- wood; 2- cook stove; 3- cooking pot; 4- Extraction booth; 5- Fume hood; 6- Fumes 7- Gas analyser probe; 8- Gas analyzer; 9- Analyzer stand).

The stove model 4 has no chimney and is made of clay with a thermal conductivity of 0.25 W/mK. The stove weight is 28.8 kg. The grate was made of clay with top radius of 7 cm, which was cut to fit into the stove. Holes were punched in the cut sheet of approximately 10 mm diameter. Two selected commonly available tree species [*Delonix regia* (Dorawa) and *Cassia siamea* (Kadai)] within the region of this research work and most commonly used as fuel wood for household cooking and heating were used (all wood fuels included the original bark content). The fuels were combusted applying the same burning conditions, designed to mimic normal kitchen or domestic cooking conditions. Table 1 gives the characteristics of the wood species. An extraction booth with the top being conical to the vertex to allow free draft for the collection of smoke from the stove was constructed (see Fig. 1 for schematic). The entire stove and the pots were enclosed in an extraction booth. Flue gases were removed from the hood through a duct of 200 mm diameter. It was crucial to conduct laboratory experiments in a location with the fume extraction system so as to carry the flue gases away from the chimney of the stove and subsequently expunge any combustion gases which may leak. The height of the chimney ensured the buoyancy needed to extract air from the surrounding air and from burning gases through the stove. The tests were carried out over a one-hour cycle using about 2 kg of the various wood species fed in at regular intervals. The real time testing was aimed to assess the catalyst activity in the event of a possible deactivation or catalyst scale off with regards to a lengthy exposure time (through a 3-time repetition of the one hour test duration). The duct was then connected through the gas-sampling probe into the Master Nanhua Portable Automotive Exhaust Gas Analyzer (Model, NHA-506EN). Each set of the CO emission experiment was conducted three times and the average readings were taken. The carbon dioxide (CO_2_) present in the exhaust gas was determined to verify the condition of combustion.

**Table 1:** Wood specie parameters (Kulla, 2011).

Description	1	2
Name	*Delonix regia*	*Cassia siamea*
Family name	Caesalpinioideae	Caesalpinioideae
Local name	Dorawa Turawa (barkachi)	Kadai
Calorific value for wood	24.34	29.03
Calorific value for charcoal	38.4	35.08

## 3 Results and discussion

**Fig. 2:**
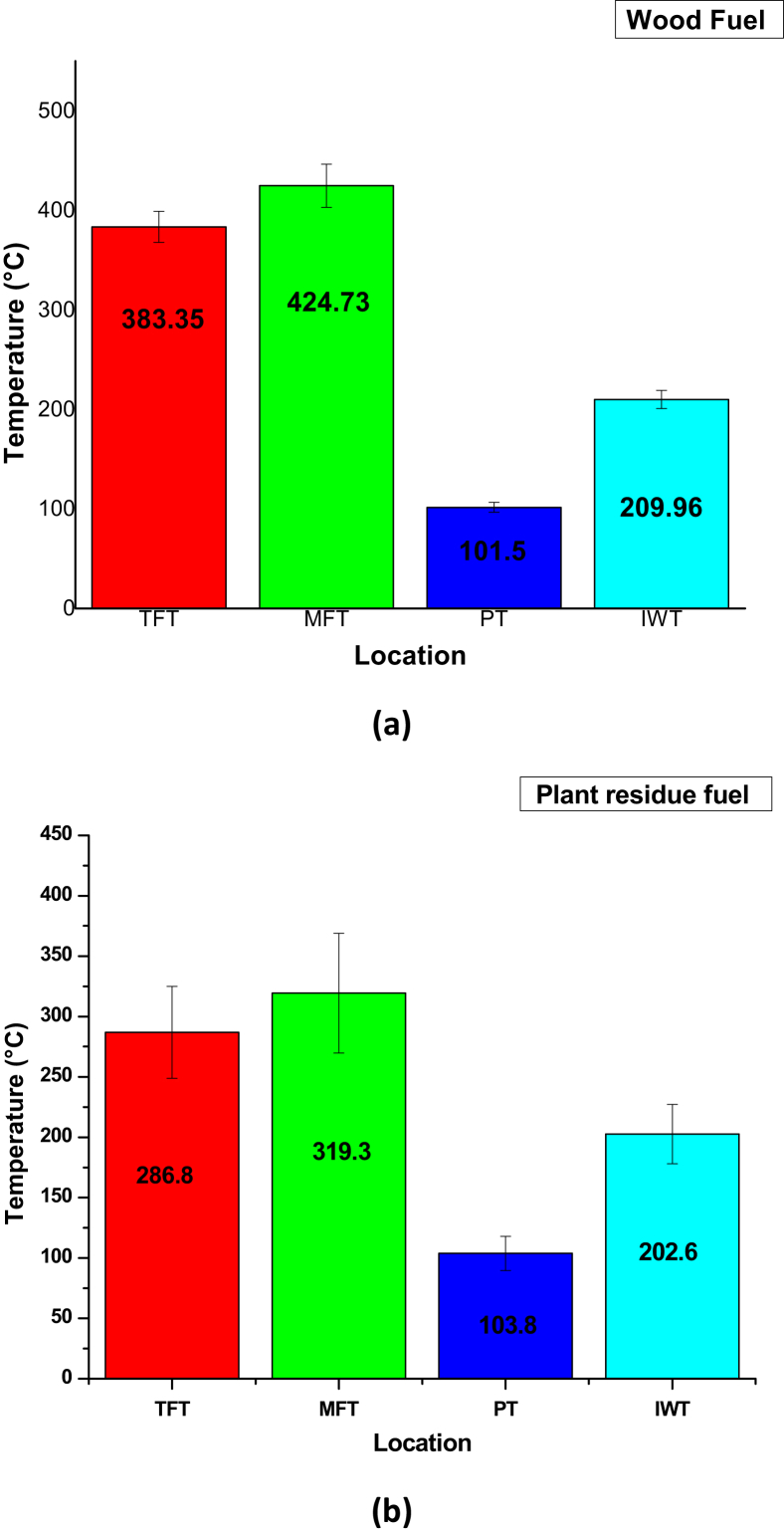
Temperature measurement data for cook stoves: (T.F.T – top flame temperature; M.F.T – middle flame temperature; P.T – pot temperature; I.W.T – inner wall temperature). (a) fuel type, wood; (b) fuel type, plant residue.

The temperature data collected for the several points in the cook stoves during the survey is shown in Fig. 2(a)–(b) with their associated error bars. The margin of error is relatively considerable, however comparatively, errors noticed for measurements using plant residue as fuel can be ascribed to the reduced sample size. The results show that the temperature of these points is in the range of 203–425 °C (albeit the pot temperature) and temperatures within this range are suitable for the physico-chemical properties of the catalysts during operation.

The metal composition of the parent material (zeolite Y) and the metal loaded zeolites are shown in Table 2. The metal loaded zeolites (MeOx-ZY: Me = Co, Fe, Co-Fe) showed a reduction in Si/Al ratio as compared to the parent material because of the presence of the metal oxides in the lattice of zeolite supports. In addition, different types of metal oxides (e.g. CaO, MgO, TiO_2_) etc, were detected in the XRF spectrum of parent material and metal loaded zeolites which shows that the catalyst samples consist of different physicochemical properties. It is worthy of note that the Co/ZY catalyst did not show an appreciable difference in silica to alumina ratio because the instrument could not detect the cobalt metal. However, Fe/ZY showed an appreciable decrease in Si/Al ratio because the ferrite metal composition (8.276 wt %) in the lattice was significant. Interestingly, by observing the Co-Fe/ZY catalyst which had an equal amount of cobalt and iron loadings, and by reason of the instruments' inability to capture cobalt, it can be noticed that the elemental composition of iron loading was half as compared to Fe/ZY catalysts. This is a strong indication of the accuracy in terms of elemental composition of the parent material and synthesized catalysts.

**Table 2:** Chemical compositions of the Zeolite Y and modified zeolites.

Component	SiO_2_	Al_2_O_3_	Fe_2_O_3_	TiO_2_	MgO	CaO	K_2_O	Cl	Si/Al	LOI*
ZY (wt%)	96.404	3.226	0.012	0.023	0.090	0.014	0.015	0.013	50.2	0.06
Co/ZY (wt%)	96.293	3.289	0.031	0.027	0.083	0.005	0.025	0.005	50.1	0.24
Fe/ZY (wt%)	88.235	3.129	8.276	0.035	0.091	0.028	0.016	0.008	47.5	0.18
Co-Fe/ZY (wt%)	91.938	3.156	4.565	0.033	0.075	0.039	0.017	0.006	49.1	0.17

LoI* = Loss on ignition; ZY = zeolite Y; Co/ZY = cobalt/zeolite Y; Fe/ZY = iron/zeolite Y; Co-Fe/ZY = cobalt-iron/zeolite Y; Si/Al; = silica to alumina ratio.

**Fig. 3:**
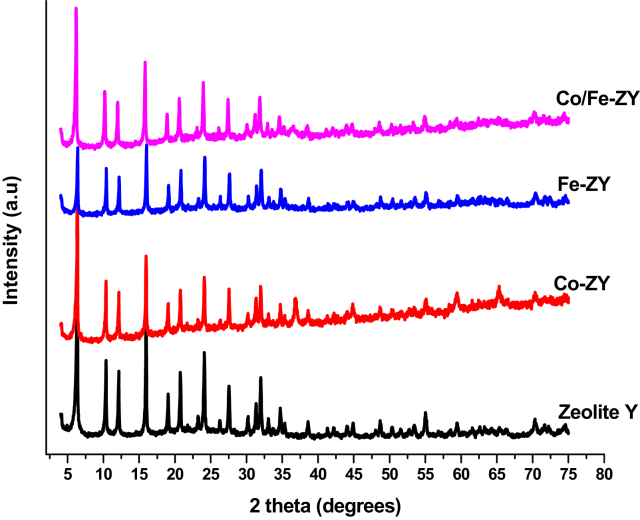
XRD patterns for parent material and synthesized catalysts.

As shown in Fig. 3 the zeolite Y and metal loaded zeolites (Co, Fe, Co-Fe/ZY) show reflections at 2θ: 6.2, 10.1, 12, 15.8, 20.2, 23.5, and 31 which is in line with reflections of zeolite Y in literature (Robson, 2001). Generally, all the samples tested showed high crystallinity. It means all the metal loaded zeolites maintained the usual crystal reflections of Zeolite Y, suggesting that the crystal structure of the zeolite was not destroyed by the loading of the metal precursors and thermal treatment (Li et al., 2016). Co-based catalysts exhibited the characteristic reflections of Co_3_O_4_ at 2θ of 37° due to the dissociation of Co(NO_3_)_2_ during calcination in air (Sun et al., 2016). No diffraction peak for Fe_2_O_3_ can be found in Fe/ZY or Co-Fe/ZY. This means that iron metal species highly dispersed on the external surface of the zeolite which makes it hard to detect (Li et al., 2016; Cheng et al., 2017).

**Fig. 4:**
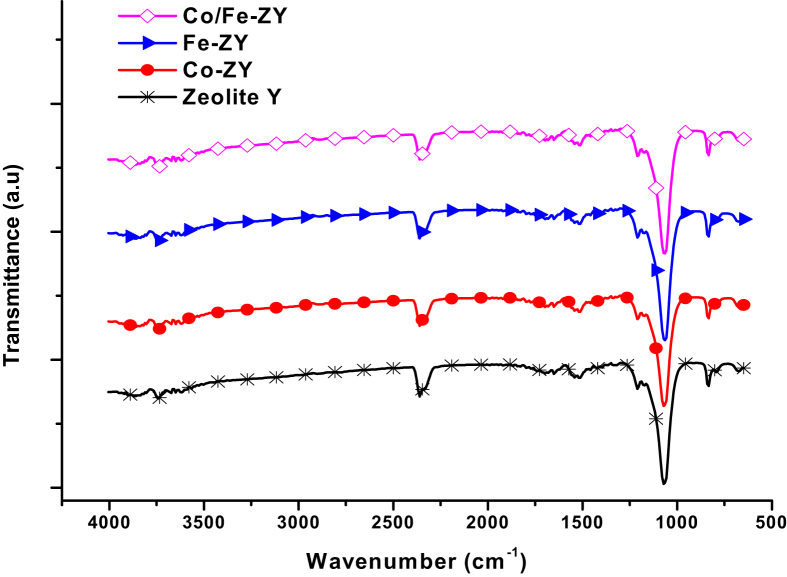
FTIR spectra for parent material and synthesized catalysts.

The FTIR spectra of zeolite-Y and the metal loaded zeolites as shown in Fig. 4. The spectra is mainly dominated by the strong zeolite bands (Jin et al., 2006). As seen, the typical peaks of zeolite Y support are not destroyed while modifying with mono/bimetal precursors.

**Fig. 5:**
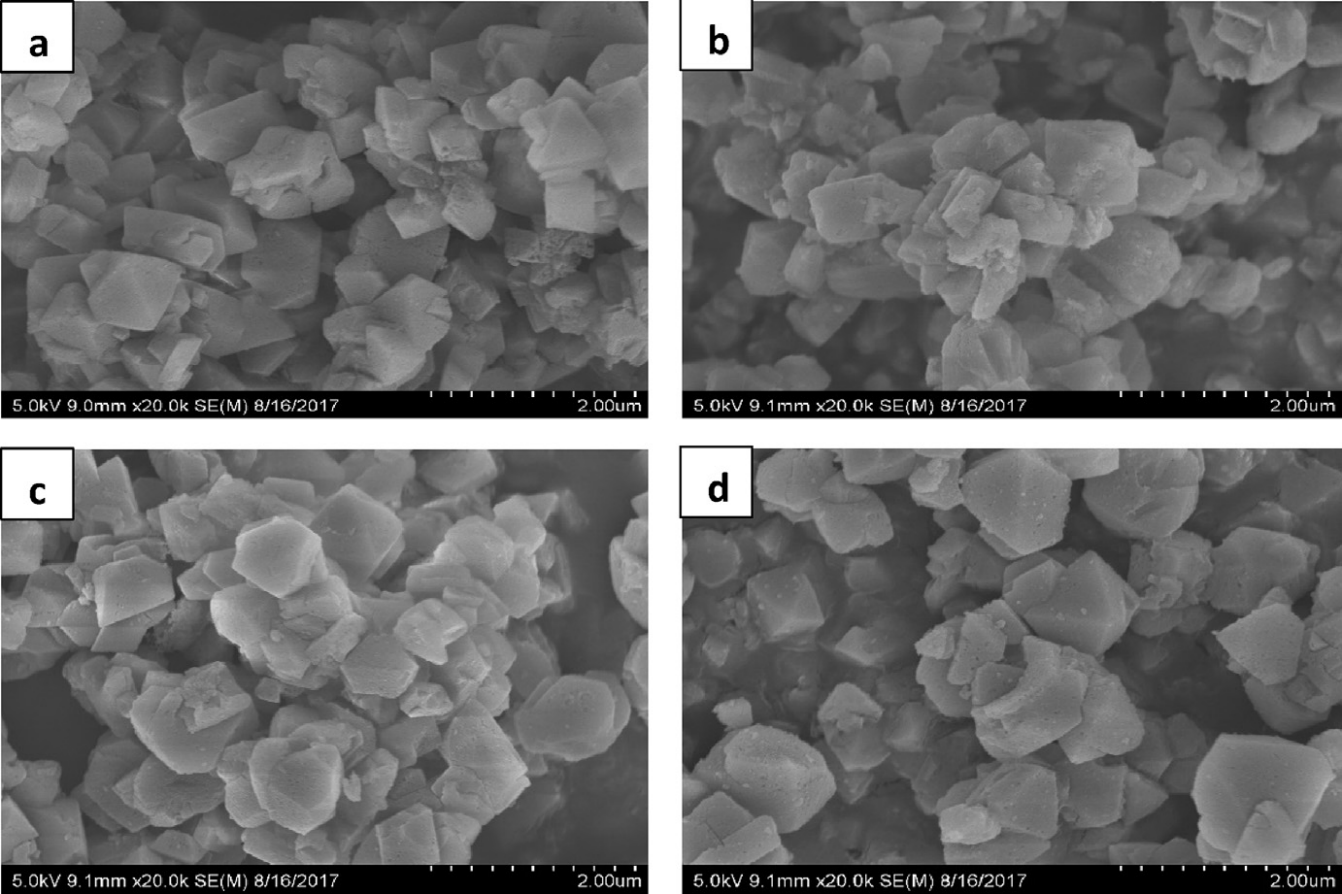
SEM images of parent material and synthesized catalysts: (a) Zeolite Y, (b) Co/ZY, (c) Fe/ZY, (d) Co-Fe/ZY.

SEM images of the samples collected are presented in Fig. 5. Similar morphologies with Zeolite Y were observed and showed some agglomeration which can be due to the interconnectivity of the particles (Vichaphund et al., 2014). All the samples showed relatively uniform spherical particles in the size range of 2 μm. SEM images further revealed a uniform particle size of all the samples with a regular shape that indicates that the zeolite crystallites were not affected by the Co, Fe and Co-Fe loadings. We presume that the sintering temperature (550 °C) was beneficial to the crystal growth.

**Fig. 6:**
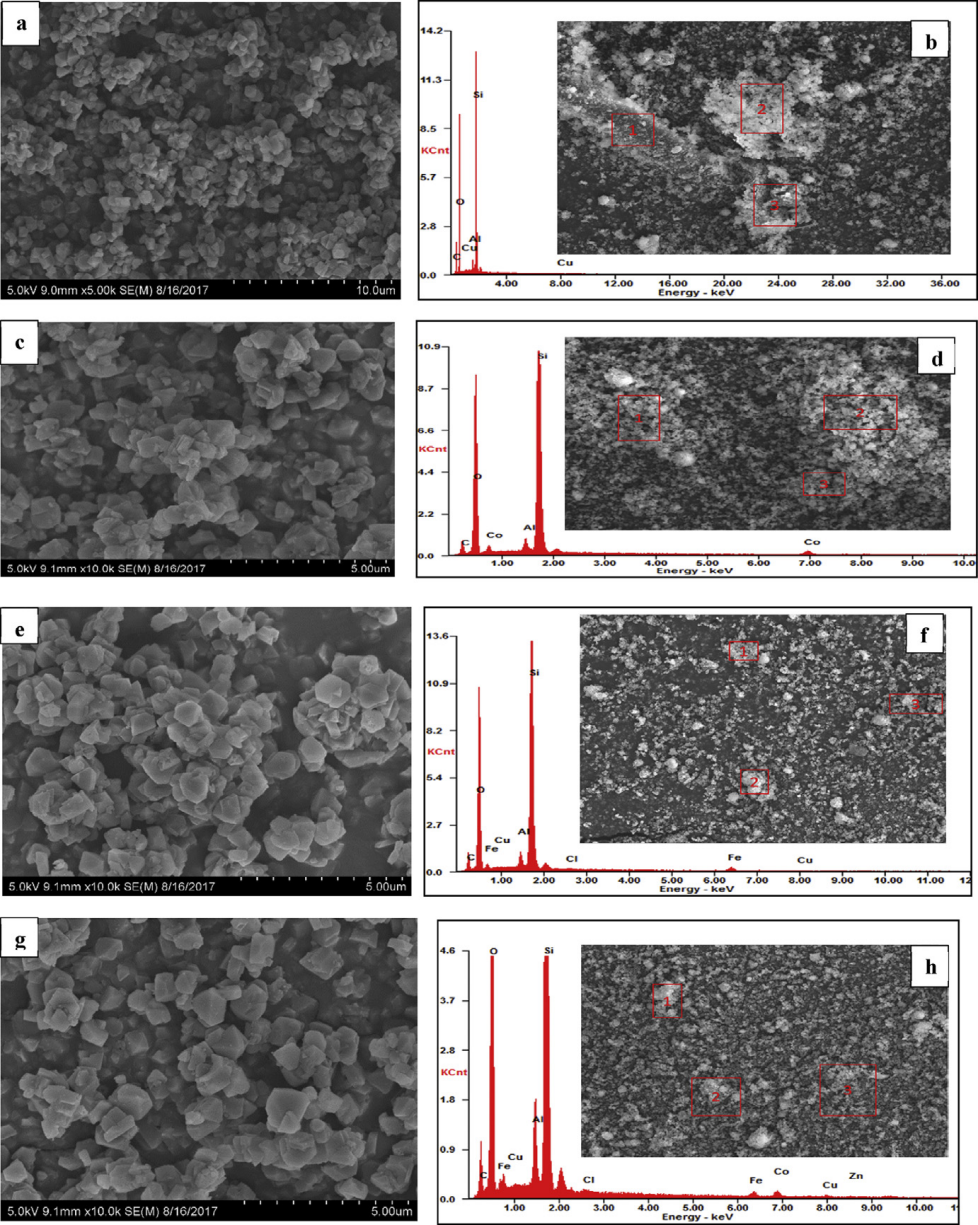
SEM/EDS of parent material and synthesized catalysts: (a) ZY, (b) ZY-EDS, (c) Co/ZY, (d) Co/ZY-EDS, (e) Fe/ZY, (f) Fe/ZY-EDS, (g) Co-Fe/ZY, (h) Co-Fe/ZY-EDS.

Typical SEM and energy-dispersive spectroscopy (EDS) analysis of the parent zeolite Y and MeOx-ZY are presented in Fig. 6. The EDX results collected from different points give similar compositions and indicates that Co-ZY was composed of Co, Si, Al, and O (the C species were caused by the conductive plastic), Co-Fe/ZY was composed of Co, Fe Si, Al, and O, and Fe-ZY was composed of Fe, Si, Al, and O. The spectrum also shows minor elements such as Cl, Cu, and Zn which were due to contamination.

The BET surface area, Langmuir surface area, and pore volume data of zeolite-Y and MeOx-ZY are presented in Table 3. The possibility of the destruction of the zeolite-Y framework on ion-exchange can also be ruled out by the fact that only negligible reduction in the surface areas and pore volume were found upon ion exchange into the zeolite-Y framework. In addition, the surface areas and pore volumes are much lower in the case of the corresponding metal exchanged zeolite-Y. This reduction can be attributed to the filling of the zeolite-Y nanopores with transition metal complexes. This assertion is in line with other researchers (Balkus and Gabrielov, 1995; Varkey and Jacob, 1998; Gong et al., 2017).

**Table 3:** BET surface analysis of zeolite-Y and metal loaded zeolites.

Sample	BET surface area (m^2^/g)	Langmuir surface area (m^2^/g)	Pore volume (cm^2^/g)
ZY	642.33	857.90	0.766825
Co/ZY	557.57	742.33	0.532925
Fe/ZY	548.00	720.20	0.280181
Co-Fe/ZY	482.54	656.77	0.265633

**Fig. 7:**
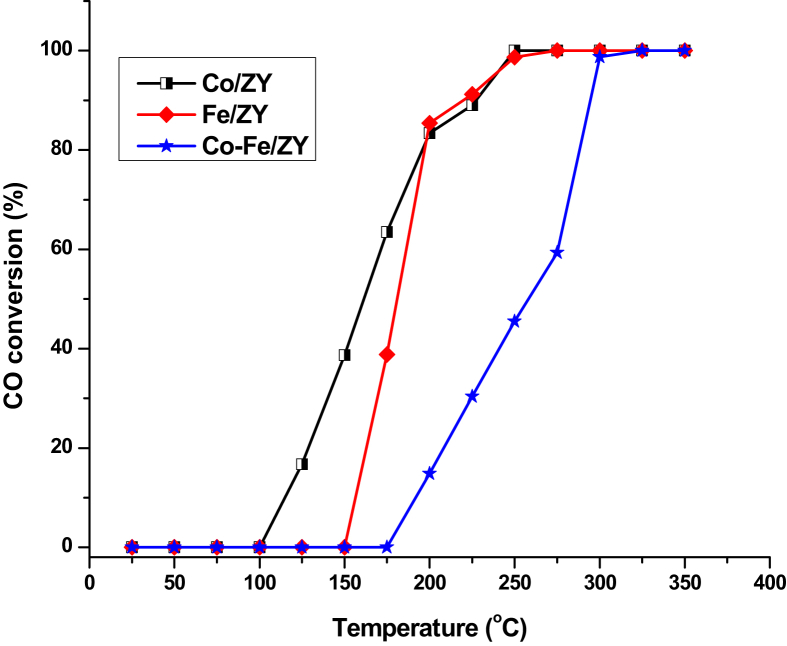
CO oxidation over zeolite based catalysts at 20000 h^−1^ space velocity.

Fig. 7 shows CO oxidation activity for all synthesized catalysts (metal loaded zeolites). From the results, Co, Fe, and Co-Fe/ZY catalysts gets activated at about 125, 175 and 200 °C respectively. The Co/ZY catalyst shows approximately 100% CO conversion (T_100_) at 250 °C for initial CO concentration of 1000 ppm, while T_100_ was increased to 275 °C and 325 °C for Fe/ZY and Co-Fe/ZY catalyst respectively at space velocity of 20000 h^−1^. The most promising activity of Co/ZY can be attributed to the ability of CoO to be present at multiple or stable oxidation states to make it more efficient in generating reactive oxygen species (Leocadio et al., 2004) without any inhibition effect from bi-metallic loadings. Generally, all catalysts converted CO to CO_2_ and this is supported by the high surface area as well as the ordered and porous structure of the zeolite support facilitating mass transfer. These results are similar to comparatively higher activity recorded for zeolite based catalysts with the incorporation of precious metals reported by (Lokhande et al., 2015).

**Fig. 8:**
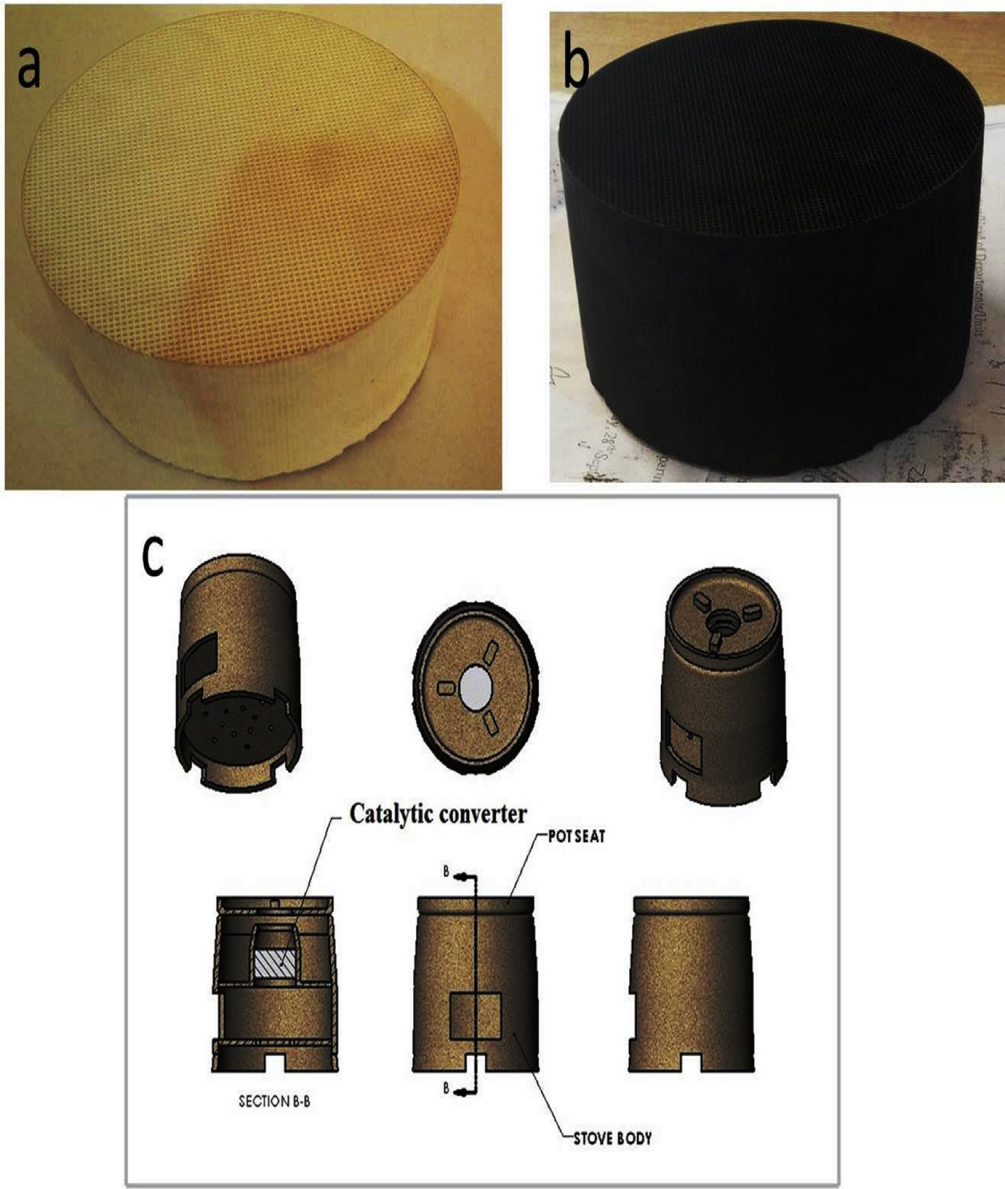
Illustrations of: (a) bare ceramic support (b) coated ceramic substrate (c) views of the assembly of the prototype stove.

The bare ceramic and the coated ceramic substrates (coated with the best catalyst {Co-based catalyst}) are shown in Fig. 8. The change in colour for the coated ceramic substrate is ascribed to oxidation of the metal oxides thereby changing the surface chemistry. The views of the prototype traditional stove used in this study and the incorporation of the catalytic converter in the stove are also presented in Fig. 8c.

**Fig. 9:**
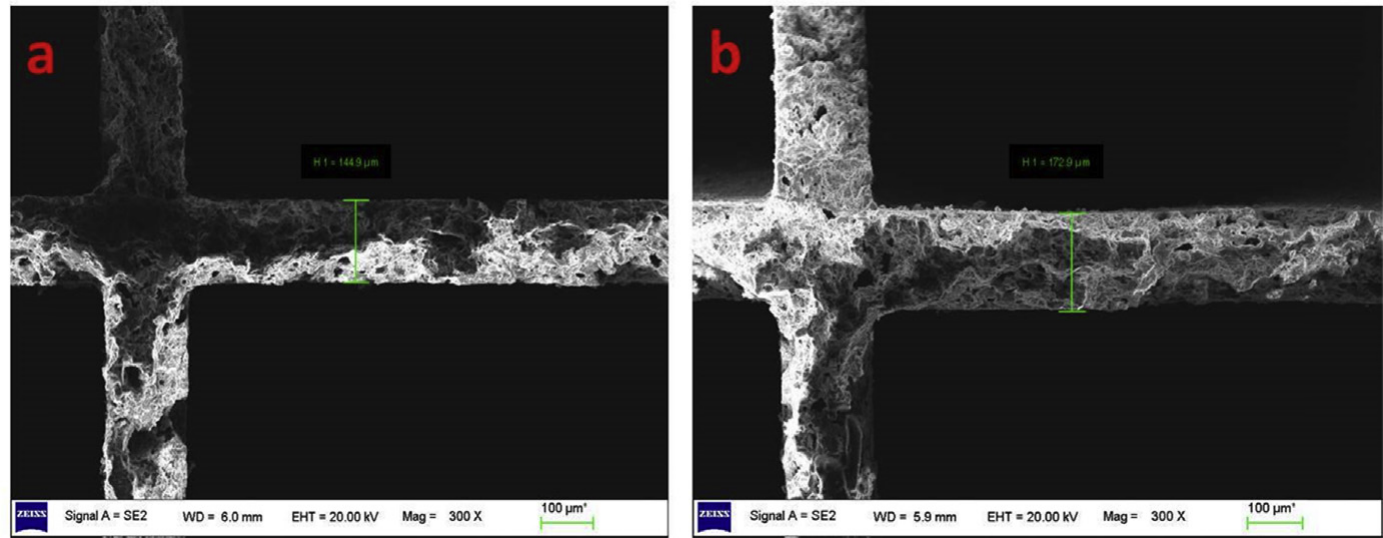
SEM images for coating morphology of catalytic converter: (a) bare ceramic substrate (b) coated ceramic substrate including cross-sectional interface structure of catalyst coating and honeycomb.

The SEM images of the bare and coated ceramic substrates, which include catalyst cross sections, are shown in Fig. 9. From Fig. 9a & b, it can be seen that a uniform coating on the ceramic substrate is formed with a thickness of about 144.49 μm and 172.9 μm respectively. This interface on the coated ceramic usually results in more reliable adhesion in catalyst coatings. This basically prevents catalyst coatings from scaling off from supports during operation.

**Fig. 10:**
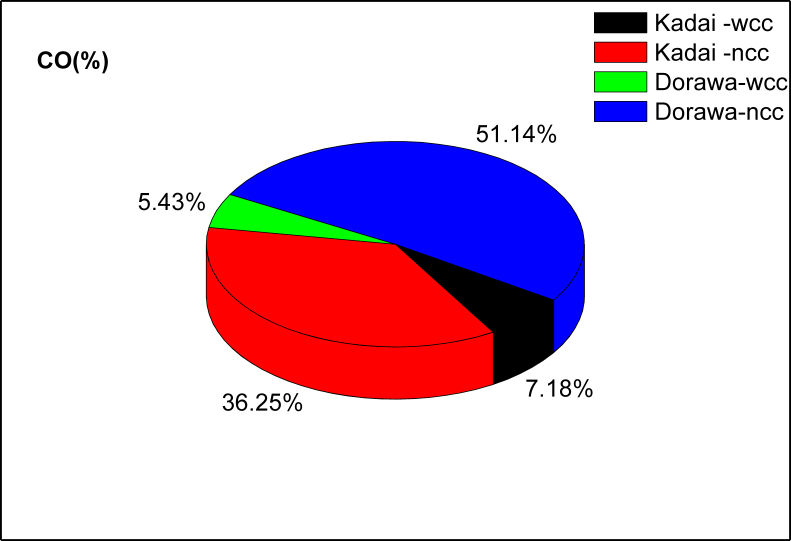
CO emission plots for the combustion of wood species.

The percentage CO emission without catalytic intervention (ncc) for the Dorawa and Kadai wood species were 36.25 and 51.24% respectively (see Fig. 10). This implies a larger CO emission for the Dorawa wood under the same conditions. High volatile fuels such as wood should yield a low CO emission which is expected in a combustion device that has enough oxygen and temperature. The higher CO emission released from this set-up can be related to the combustion experiments which mimics real life domestic cooking. The increase in CO emissions can also be related to higher carbon content and subsequently higher heating value for the Dorawa wood. The CO emission reduces significantly with the incorporation of the catalytic converter (wcc). The values obtained were 5.43 and 7.18% for Dorawa and Kadai wood species respectively. This reduction can be ascribed to oxygen assisted combustion through the dissociation of NOx by providing more oxygen which increases air supply to the stove. When air flow through the combustion zone is enhanced, it increases the combustion efficiency and vice-versa (Medina et al., 2017).

**Fig. 11:**
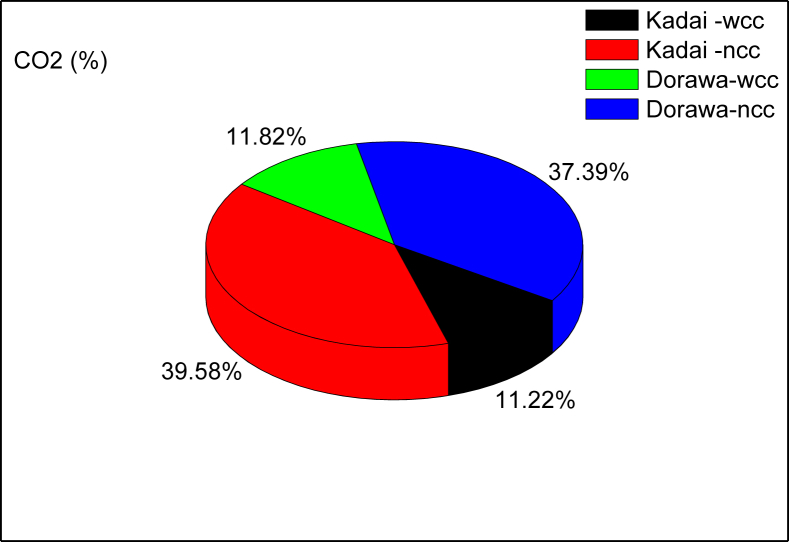
CO_2_ emission plots for the combustion of wood species.

With catalytic intervention (wcc), values of CO_2_ obtained were 11.82 and 11.22% for Dorawa and Kadai wood species respectively (see Fig. 11). These values can be regarded as acceptable considering its effect on users of the combustor. Without the catalytic converter (ncc), 37.39 and 35.58% of CO_2_ was released for Dorawa and Kadai wood species respectively. The initial rise in CO is accompanied by a huge release of O_2_ which assists in the combustion process. The surface redox reactions which enables the catalysts light up, with progressive increase in temperature is hugely consequential for the improved combustion as observed. In this way sufficiently high temperatures should be maintained for the catalysts to perform effectively.

## 4 Conclusions

This study investigated the catalytic abatement of CO species from incomplete combustion of specific wood species commonly used for domestic cooking in northern Nigeria.

Results from the temperature data collected from different stove locations (TFT, MFT, PT, IWT) sampled from 50 commercial and household cook stoves indicate that the temperature range (203–425 °C) is suitable for catalytic interventions.

The CO oxidation using simulated exhaust gases in a fixed bed reactor suggests that the as-used catalysts are effective and zeolite Y is observed to be a good catalyst support. The better activity of Co impregnated zeolite catalyst (Co/ZY) for CO oxidation reactions can be attributed to the ability of CoO to be present at multiple or stable oxidation states to make it more efficient in generating reactive oxygen species without any inhibitive effects from the bi-metallic loadings. The catalytic converter performed well in terms of CO abatement for both wood species with observed CO emissions being slightly higher from Kadai than Dorawa wood. Based on the obtained results, Dorawa wood is recommended for use in the areas of interest based on the emissions trade-off.

## Declarations

### Author contribution statement

David O. Obada: Conceived and designed the experiments; Performed the experiments; Wrote the paper.

Mary Peter: Conceived and designed the experiments; Performed the experiments.

Dangana M. Kulla: Conceived and designed the experiments.

Nua O. Omisanya, David Dodoo-Arhin: Analyzed and interpreted the data; Wrote the paper.

Abdulazeez Y. Atta: Analyzed and interpreted the data.

### Funding statement

This research did not receive any specific grant from funding agencies in the public, commercial, or not-for-profit sectors.

### Competing interest statement

The authors declare no conflict of interest.

### Additional information

No additional information is available for this paper.
